# Supplementary figures and images for: Developmental Methylome of the Medicinal Plant Catharanthus roseus Unravels the Tissue-Specific Control of the Monoterpene Indole Alkaloid Pathway by DNA Methylation
Source: Int J Mol Sci. 2020 Aug 21;21(17):6028. doi: 10.3390/ijms21176028 (PMC7503379; doi:10.3390/ijms21176028)

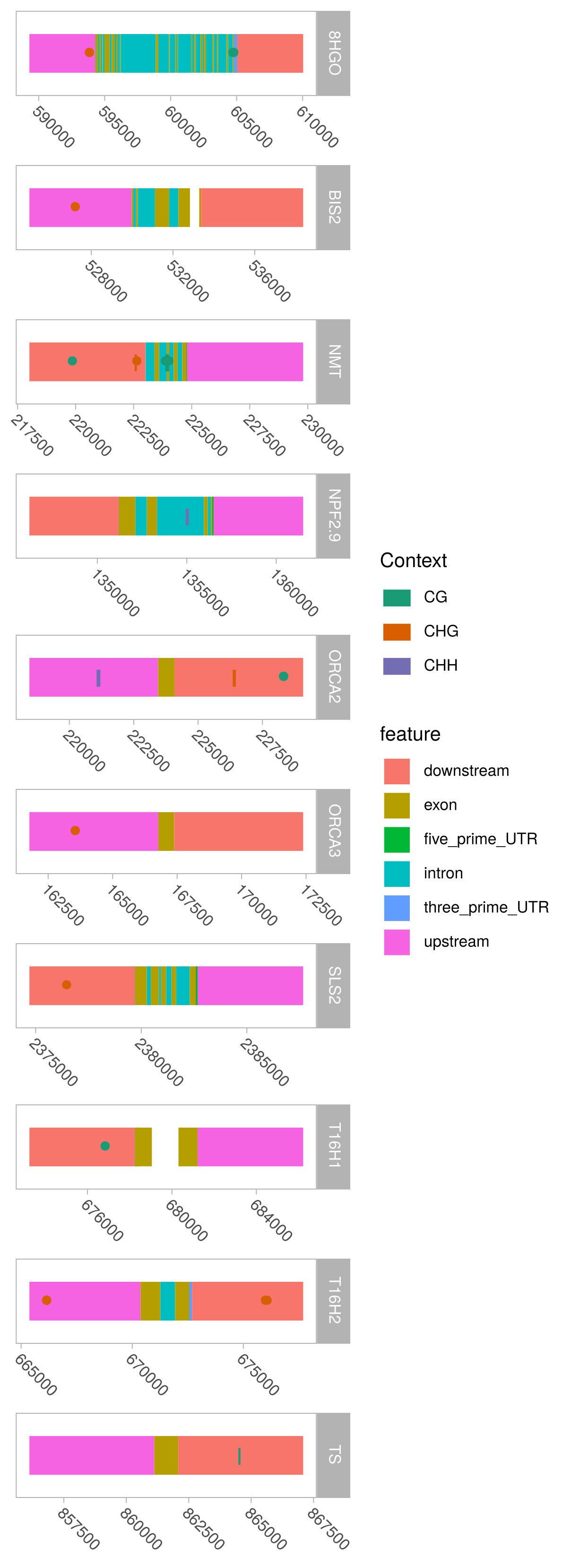

Supplement: Supplementary file 1 [file ijms-21-06028-s001.zip › supplementary_material/SupplFig6.png]

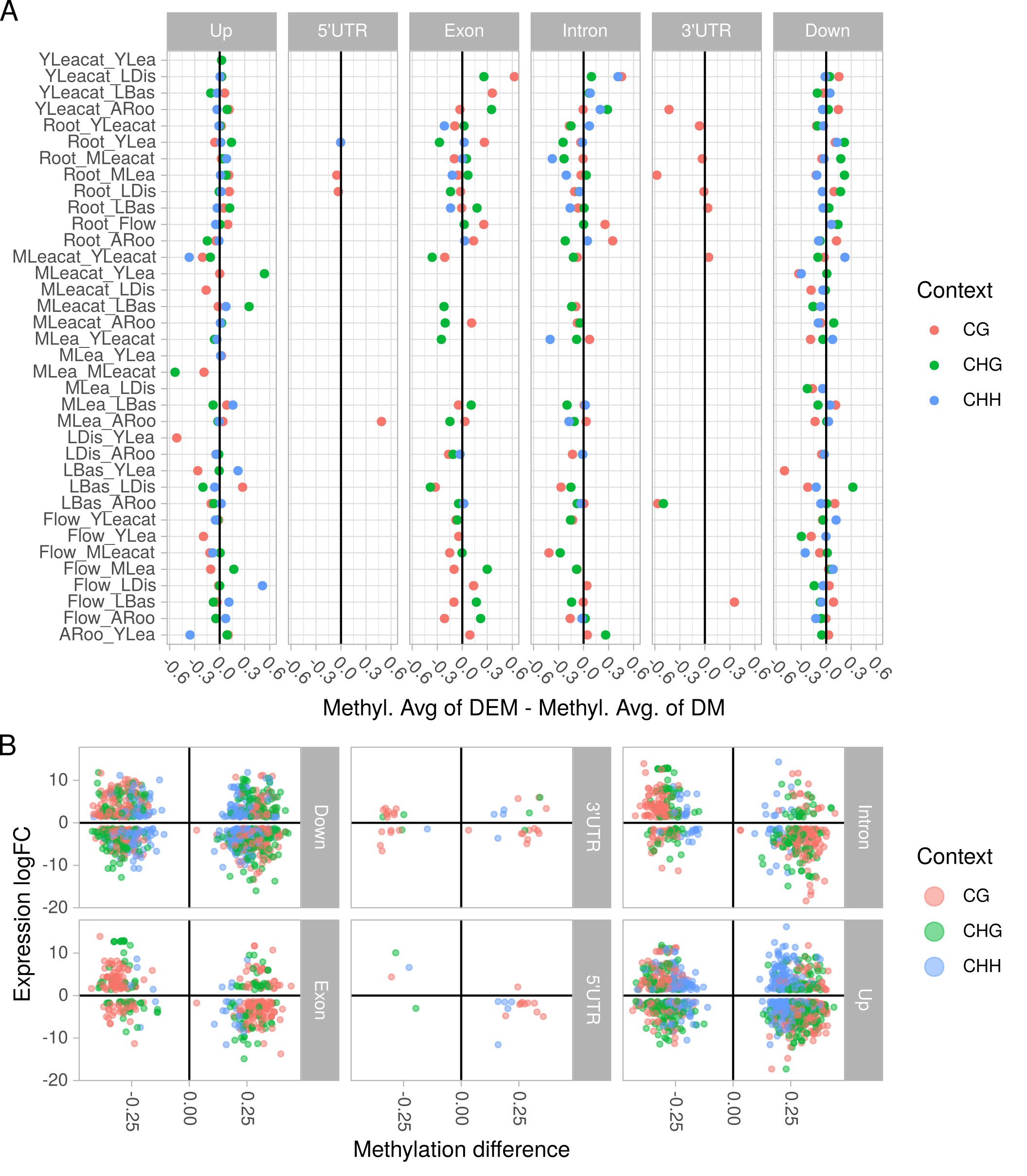

Supplement: Supplementary file 1 [file ijms-21-06028-s001.zip › supplementary_material/SupplFig3.png]

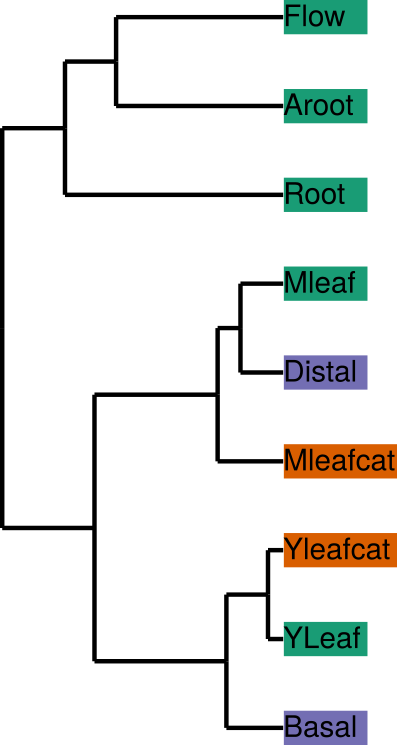

Supplement: Supplementary file 1 [file ijms-21-06028-s001.zip › supplementary_material/SupplFig2.png]

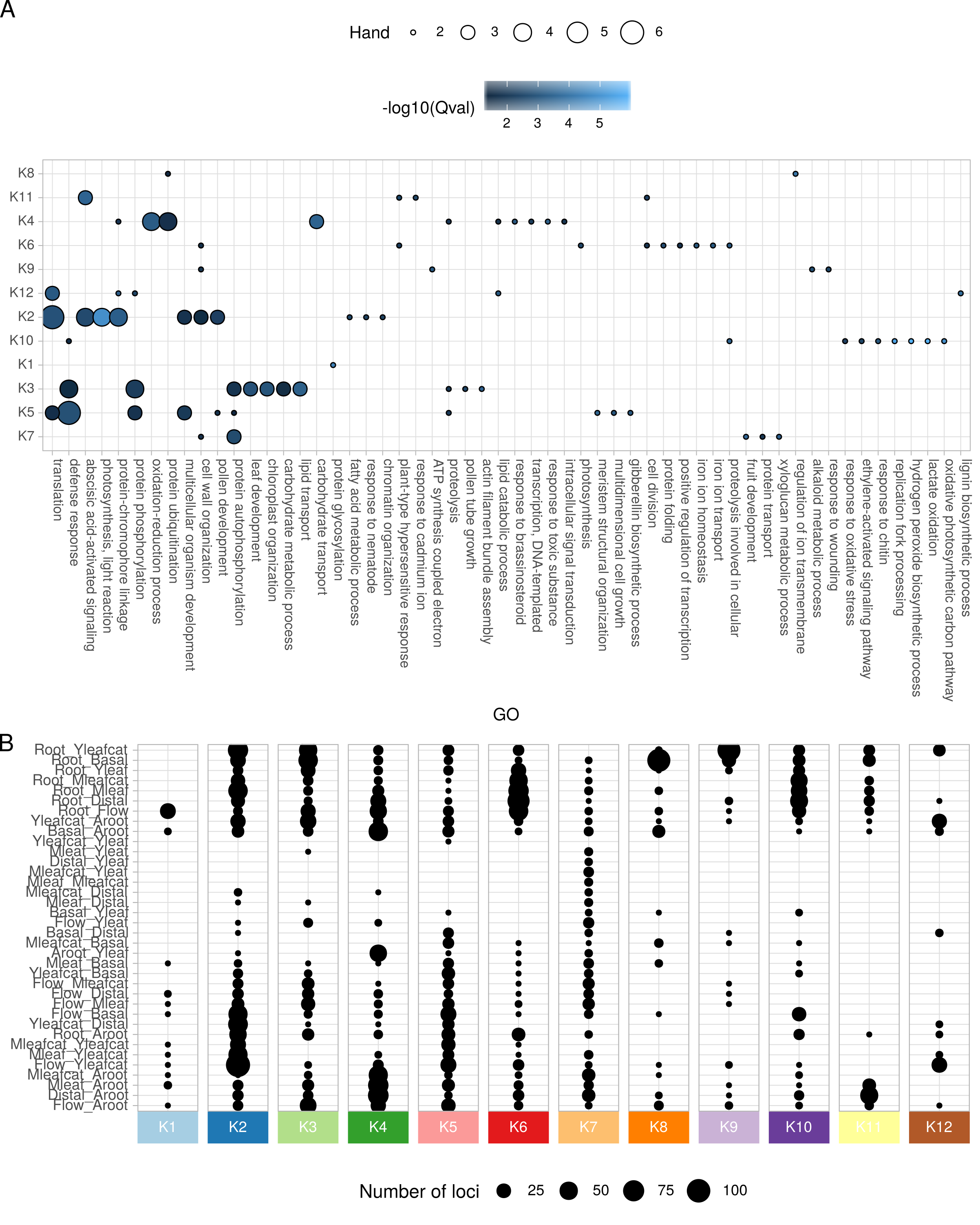

Supplement: Supplementary file 1 [file ijms-21-06028-s001.zip › supplementary_material/SupplFig5.png]

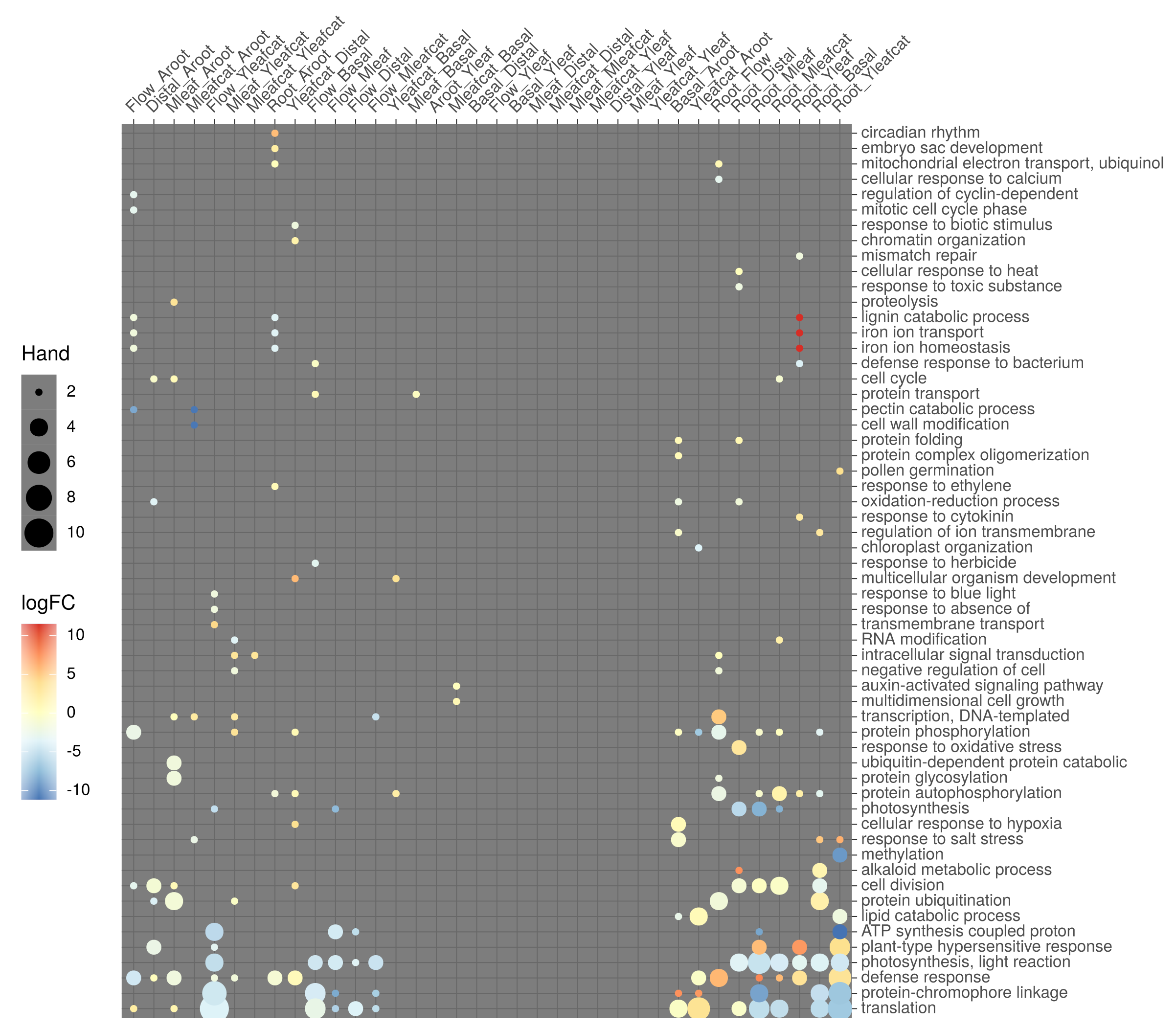

Supplement: Supplementary file 1 [file ijms-21-06028-s001.zip › supplementary_material/SupplFig4.png]

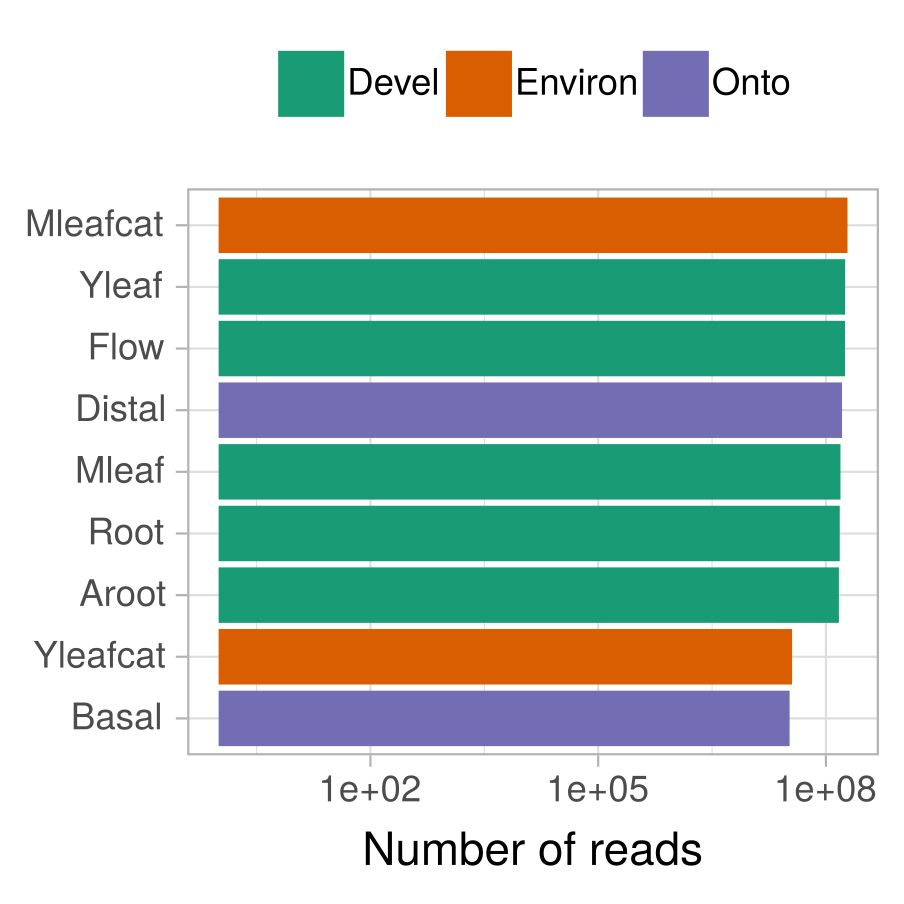

Supplement: Supplementary file 1 [file ijms-21-06028-s001.zip › supplementary_material/SupplFig1.png]
